# 

**Published:** 1905-02-04

**Authors:** 


**NOTICE TO CORRESPONDENTS.**

All MSS., Letters, Books for Review, and other matters intended for the Editor should be addressed The Editor, "The Hospital." The Hospital Buildings, 28 & 29 Southampton Street, Strand, W.C.

The Editor cannot undertake to return rejected MS., even when accompanied by stamped directed envelope.

**Notice to Advertisers and Subscribers.**

All Advertisements, Orders for Copies of The Hospital, and Business Communications should be addressed to **THE MANAGER (Not to the Editor),** The Hospital, 28 & 29 Southampton Street, Strand, London, W.C.

The Cost of Subscription, post free, is as follows:—Per week, 3½d.; per quarter, 3s. 3d.; per half-year, 6s. 6d.; per annum, 13s. Foreign and Colonial:—Per annum. 19s. 6d., payable, in all cases, in advance.

**NOTICE.—Vol. XXXVI. of "The Hospital" (April to September 1904), in handsome cloth binding, is now on sale at the office of this Journal, price 10s. 6d. Cases for binding the Numbers contained in this Volume 2s. Index to Vol. XXXVI., 3½d., post free.**

**The Monthly part for February is now ready. Price 1s., by post, 1s. 3d.**

**NOW READY.**

### AN ELEMENTARY TREATISE ON THE LIGHT TREATMENT FOR NURSES (Illustrated)

**2/6 net.**

**2/9 post-free.**

By JAMES H. SEQUEIRA, M.D.

**Physician in charge of the Skin Department and Lecturer on Dermatology at the London Hospital.**

CONTENTS.

Introduction.
Chapter I.—Light.
Chapter II.—The Influence of Light upon Living Organisms.
Chapter III.—The Treatment of Small-pox by Red Light.
Chapter IV.—The Treatment of Lupus by Light.
Chapter V.—The Apparatus for Treatment by the Chemical Rays of Light.
Chapter VI.—The Technique of the Finsen Treatment.
Chapter VII.—Other Lamps used in the Light Treatment.
Chapter VIII.—The Diseases Treated by the Chemical Rays of Light.

### CASE BOOKS AND CHARTS.

**A CASE BOOK FOR PRIVATE NURSING.** Arranged for Day and Night Nurse's Report, with rulings in accordance with what has been found in practice to be the most suitable and approved System for Pay Hospitals, Nursing Homes, Private Nurses, etc. The size of an Exercise Book, $7\frac{1}{2}$ in. by $9\frac{1}{2}$ in., containing 48 pp. (Special quotations for a quantity.) — **3d.** net, **4½d.** Post free.

**THE "SIMPLEX" DISTRICT NURSES' CASE BOOK.** Containing space for 50 cases, ruled and printed, bound in cloth boards. — **6d.** net, **7d.** Post free.

**THE POCKET CASE BOOK FOR DISTRICT AND PRIVATE NURSES.**

By a Physician.

Demy 16mo, cloth boards . . . **1/-** Post free.

In handsome leather, gilt . . . **1/6** net, **1/7** Post free.

**DISTRICT MIDWIFERY CASE BOOK.** Compiled by Margaret Caley, late Matron of Fulham Midwifery Training School. — **6d.** net, **7d.** Post free.

Suitable size for the Pocket.

**POCKET-BOOK OF TEMPERATURE CHARTS.** Containing 17 Twelve-hour and 8 Four-hour Charts, perforated so that each Chart may be removed after use. — **6d.** net, **7d.** Post free.

**TEMPERATURE CHARTS, MORNING AND EVENING.**

Size 10 in. by 8 in.

Carriage paid.

| | | | |
|---|---|---|---|
| 25 . . | **1/-** | 250 . . | **6/6** |
| 50 . . | **1/6** | 500 . . | **12/-** |
| 100 . . | **2/9** | 1,000 . . | **23/-** |

**JAPANNED TIN WASHABLE CHART-HOLDERS.**

Each . . . . . . **1/-**

Per dozen . . . . . **9/6**

COMPLETE CATALOGUE OF NURSING MANUALS POST FREE ON APPLICATION.

### THE SCIENTIFIC PRESS, LTD.,

**28 & 29 SOUTHAMPTON STREET, STRAND, LONDON, W.C.**

